# A Narrative Review to Identify Promising Approaches for Digital Health Interventions to Support Emotion Regulation for Adolescents With Attention-Deficit/Hyperactivity Disorder

**DOI:** 10.2196/56066

**Published:** 2025-02-27

**Authors:** Aja Louise Murray, Melissa Thye, Ingrid Obsuth, Shufang Cai, Michael Lui, Corina Orr, Anusha Saravanan

**Affiliations:** 1 Department of Psychology University of Edinburgh Edinburgh United Kingdom; 2 Health in Social Science University of Edinburgh Edinburgh United Kingdom

**Keywords:** attention-deficit/hyperactivity disorder, ADHD, digital health intervention, adolescence, emotion regulation, emotion dysregulation, mobile phone, emotion, teens, youths, narrative review, support, development, design, regulation, young people, evaluation, neurodiversity, neurodivergent, attention deficit, neurodiverse, neuroscience, mental health, digital mental health

## Abstract

Emotion regulation difficulties affect many adolescents with attention-deficit/hyperactivity disorder (ADHD), and previous research has highlighted a need for accessible interventions to support them in this domain, especially in real-life contexts. Digital health interventions (DHIs) can be embedded in adolescents’ daily lives and thus offer considerable promise for meeting this need. However, there is a lack of information to guide the development of suitable emotion regulation DHIs for this population. The goal of this study is, therefore, to identify recommendations to guide the development of emotion regulation DHIs for adolescents with ADHD. This narrative review synthesizes diverse relevant evidence to inform their development, including promising therapeutic approaches and components and relevant design and development considerations. We find that there is very little direct evidence of “what works” for emotion regulation DHIs and emotion regulation interventions more generally for adolescents with ADHD; however, we identify promising therapeutic approaches for new DHIs. We also recommend following a co-design or coproduction approach with adolescents with ADHD, including exploring elements designed to motivate and engage young people to support sustained adherence. We conclude that DHIs are a promising approach for emotion regulation interventions for adolescents with ADHD, could draw on a range of existing therapeutic approaches, and should be co-designed with users themselves.

## Introduction

Emotion regulation difficulties are commonly experienced by adolescents with attention-deficit/hyperactivity disorder (ADHD) and are thought to contribute to frequently co-occurring issues, such as anxiety, depression, and conduct problems [1,2]. However, existing interventions for emotion regulation in adolescents with ADHD have important limitations, including poor generalizability to real-life situations, not addressing the linkages between emotion regulation and core areas of impacted functioning such as peer problems, and lacking a coproduction approach that can be essential for ensuring that the intervention truly meets the needs of its target population [3-5]. As such, there remains a need for new accessible interventions to support emotion regulation and associated functioning among adolescents with ADHD, especially those that can support the application of emotion regulation skills in real-life contexts. Interventions that are “ecologically” embedded, facilitating emotion regulation generalization in daily life are particularly needed. As smartphone-delivered digital health interventions (DHIs) have a strong potential to meet this need, the purpose of this review is to synthesize the relevant evidence on the most promising intervention approaches, components, and other design and development considerations to provide recommendations for emotion regulation DHI development for adolescents with ADHD. We use a narrative review approach because of the heterogeneity of the relevant evidence base.

## Emotion Regulation in Adolescents With ADHD

Emotion regulation can be defined as a set of processes concerned with the identification or evaluation, inhibition, or modification of emotional reactions in the service of achieving some goal, including displaying socially appropriate behavior [6]. The process model of emotion regulation, developed and later extended by Gross [7,8], is one of the dominant models in the field. It categorizes strategies into 2 main types: antecedent-focused and response-focused and highlights the dynamic and context-dependent nature of how individuals regulate their emotions to adapt to various situations and personal goals. Antecedent-focused strategies involve modifying emotional experiences before they fully develop (eg, via cognitive reappraisal), while response-focused strategies involve managing emotions after they have emerged (eg, suppression). Three stages of emotion regulation are specified: identification (distinguishing emotional responses and determining whether to regulate them), selection (choosing which strategies to apply), and implementation (applying a strategy). Emotion dysregulation can manifest in a range of ways, including a failure of emotion recognition, a lack of empathy or emotional lability, overreactivity, or inertia [9]. In ADHD, emotion dysregulation is recognized as potentially including emotional reactions or experiences that are excessive relative to norms and developmental stage, uncontrolled and rapid shifts of emotion, and the abnormal allocation of attention to emotional stimuli [10]. These models suggest multiple points where successful emotion regulation can fail and correspondingly offer a range of intervention points that can be targeted with emotion regulation interventions.

Emotion regulation issues, especially emotional lability, are commonly observed among young people with ADHD and are thought to play a key role in linking ADHD to frequently co-occurring issues such as peer problems, anxiety, depression, and behavior problems [2,11-13]. For example, longitudinal research has suggested that emotion dysregulation lies on the developmental pathway between ADHD symptoms and later anxiety or depression in childhood [11]. These types of findings point to emotion regulation as a potential key target for supporting emotional functioning and reducing the risk of developing negative outcomes for young people with ADHD. However, existing interventions such as pharmacological interventions that target primary symptoms of ADHD are not as effective in reducing emotion dysregulation [3]. There are also established issues with medication adherence and discontinuation without remission in adolescence, pointing to the need for other types of interventions [14]. In addition, while there are a small number of interventions specifically targeting emotion dysregulation in ADHD, these often have limitations such as failing to generalize well to real-life situations, not being tailored for adolescence, and not adequately considering the interconnections between emotion regulation and areas of functioning such as peer relations [4,5]. Taken together, there is a need to innovate and improve interventions for emotion regulation in ADHD.

## The Promise of DHIs for Emotion Regulation in Adolescents With ADHD

It has been noted that DHIs are a promising delivery method for interventions for emotion regulation [15]. In the context of difficulties in or reluctance to access support [16,17], DHIs can make mental health interventions more accessible, may carry lower stigma, and be more attractive to young people than traditional clinical interventions [18]. In addition, many emotion regulation interventions for young people with ADHD depend on parental input [5]; however, parents can also be a barrier to accessing and engaging with mental health treatment [19,20]. For example, in one recent study, adolescents reported on the barriers they had experienced in accessing mental health treatment with 32% reporting parent-related barriers (with 8% reporting parental resistance) [20]. Though parents can be a major resource in supporting young people to engage with DHIs, they can be designed to have minimal *reliance* on parental input.

Smartphone-based DHIs in particular have become highly feasible in light of increases in smartphone ownership and use and can also offer interventions that are more embedded in people’s daily lives, with several advantages [15]. They can, for example, facilitate the gathering and use of rich data through sensors (eg, accelerometer, microphones, app use, and geolocation) or self-report (eg, symptom assessments), which help in understanding a person’s profile of strengths, weaknesses, and triggers as well as potentially in tailoring the intervention. As applied to emotion regulation DHIs, data gathered via smartphones could in principle help identify which aspects of an individual’s emotion regulation in real-life situations can benefit from intervention (eg, emotion identification, lability, reactivity, and strategy selection) and which situations or contexts are most challenging for them. Smartphone-gathered information could also be used dynamically in potential “just-in-time” adaptive interventions, in which support is provided at critical moments [21]. For example, immediate emotion regulation support could be offered if sensor or self-report data indicate that an adolescent with ADHD is experiencing escalating emotional arousal and a risk of overarousal.

Crucially, more ecologically embedded interventions may facilitate better generalization of learned skills to daily life situations by allowing users to practice skills in the flow of their daily lives. For example, though yet to be trialed in adolescents with ADHD, one recent pilot study in a population without ADHD demonstrated how smartphone apps can be used to prompt the practice of cognitive behavioral therapy (CBT) skills in real-life contexts through frequent randomly delivered prompts [22]. This study of 25 patients experiencing suicidality reported modest reductions in negative affect after being prompted to use the CBT skills they had been previously taught, suggesting that the intervention was helping to reduce symptoms in the course of daily life. Similar principles could be applied to reminding adolescents with ADHD to, for example, deploy adaptive regulation strategies taught as part of the DHI in their daily life. Such reminders may be particularly helpful in young people with ADHD, who may experience forgetfulness. Finally, DHIs can incorporate features that promote sustained engagement, such as gamification [15], discussed in more detail later.

However, despite demonstrations of the value of DHIs in other mental health contexts and their promise as a delivery method for emotion regulation interventions for adolescents with ADHD, it cannot be assumed a priori that they will be successful. There is very little direct evidence about “what works” in terms of nonpharmacological interventions for emotion regulation in ADHD and even less in relation to DHIs targeting this outcome. Even where existing successful interventions are available for emotion regulation in other populations, there is likely to be a need to adapt these for adolescents with ADHD to take account of the specific causes, manifestations, and consequences of emotion regulation difficulties in this population. In addition, their delivery should be tailored to take account of the fact that young people with ADHD may experience sustained concentration, impulsivity, introspection, and motivation difficulties that could interfere with engagement with traditionally delivered interventions. To help ensure that their future development is based on the best available evidence, we bring together existing relevant evidence on promising DHI features and therapeutic approaches that could be leveraged in effective DHIs for emotion regulation for adolescents with ADHD.

## Existing (Digital Health) Interventions Relevant for Emotion Regulation in Adolescents With ADHD

### Overview

There is limited direct evidence of the intervention approaches and components that are most promising in DHIs to support emotion regulation among adolescents with ADHD. There have been previous reviews of the evidence on DHIs for ADHD [23-26], on digital technologies for supporting emotion regulation [15,27], including for young people with developmental disabilities [28], and on emotion regulation interventions for young people with ADHD [5]; evidence bases that we summarize below. However, there is surprisingly little specific evidence on emotion regulation DHIs for adolescents with ADHD.

### DHIs for ADHD

One review examined the mobile apps for ADHD available at the time of the review and identified 109 assessment or treatment apps that met their inclusion criteria [26]. The treatment apps (n=85 focusing on treatment alone and n=11 focusing on both treatment and assessment) were based on music therapy (n=27, 28%), psychoeducation (n=23, 24%), organizational skills training (n=17, 18%), tracking medication (n=12, 13%), neurofeedback (n=8, 8%), cognitive training (n=4, 4%), and hypnosis (n=5, 5%). However, none were focused on emotion regulation in adolescents, and, importantly, the review authors noted that there was a lack of evidence supporting existing available apps.

A more recent review examined DHIs for young people with ADHD and identified 51 studies that met their inclusion criteria, covering intervention delivery via serious games or e-learning, the web, mobile health, telehealth, and augmented or virtual reality [24]. The interventions targeted domains that included cognition, socioemotional skills, behavior management, academic or organizational skills, medication adherence, vocational skills, motor behaviors, and clinical care. They found 12 well-designed and adequately powered randomized controlled trials (RCTs) reporting positive outcomes, demonstrating some promise for DHIs for young people with ADHD. For example, 1 RCT evaluating an exergaming intervention delivered via Xbox Kinect found improvements on specific executive functioning abilities (inhibition and switching reaction times) and general psychopathology in 8- to 12-year-old children with ADHD [29]. However, there was a lack of DHIs with a direct focus on emotion regulation.

A further review [23] focused on emerging technologies for supporting self-regulation in children with ADHD. In total, 36 studies met their inclusion criteria, covering a range of delivery platforms including personal computers, mobiles, virtual and augmented reality, sensors, wearable devices, and robots. Included in their review were interventions such as the EmoGalaxy video game that includes games to help children recognize, express, or regulate emotions. This was evaluated in a small-scale trial with a total of 20 children aged 7-12 years, indicating positive effects on social skills [30]. The augmented reality biofeedback intervention DEEP was also included, in which players use their breathing to control movement within a game set in an underwater environment. This was evaluated in a case study, where it was shown to reduce state anxiety [31]. However, the review highlighted the nascent stage of most DHIs in this area, with only 1 pilot RCT and most other DHIs in the development or prototyping phase. In addition, the self-regulation focus of these DHIs was generally on behavioral (eg, staying on task) rather than emotion regulation.

The most recent review in this space [28] focused on studies examining technologies for supporting emotion regulation in individuals with developmental disabilities. However, only 2 studies focused on individuals with ADHD (the EmoGalaxy and DEEP interventions discussed earlier), and the outcomes assessed in these studies were social skills and disruptive behavior, respectively; therefore, their impact on emotion regulation is not known. The authors of this review highlighted that the majority of work in DHIs for emotion regulation in developmental disabilities is focused on autism, with considerably less research on ADHD. Overall, these reviews suggest that there is a lack of DHIs for emotion regulation in adolescents with ADHD and a general preponderance of DHIs that are not (yet) supported by a strong evidence base.

### DHIs for Emotion Regulation

Given the lack of ADHD-specific DHIs, it is helpful to consider which DHIs have shown the greatest promise in other populations. Their approaches may be candidates for adaptation to adolescents with ADHD, and the lessons learned in their development and evaluation may provide relevant insights that can inform the development of ADHD-tailored DHIs. A range of reviews have examined DHIs for emotion regulation in other clinical and community populations beyond ADHD [15,27]. One [27] focused on serious games (ie, games with a therapeutic purpose) for emotion regulation in adolescence, including digital games, biofeedback, and virtual or augmented reality. An illustrative example of the reviewed serious games is MindLight: a CBT-based game drawing on biofeedback and behavior modification, which uses a survival- or horror-themed game to train children to cope with anxious feelings. In a noninferiority trial, reductions in anxiety with this intervention were comparable to those seen with CBT [32]. The review authors found that while digital games could reduce negative affect, especially in youths with anxiety, the improvement in emotion regulation was overall nonsignificant. The authors concluded that, of the approaches reviewed, digital games may have the greatest potential. Given that ADHD-specific reviews have also shown promise for digital games as a means of delivering interventions [23,24], this approach deserves further research attention as a means of engaging adolescents with ADHD in emotion regulation interventions.

A broader scoping review of “interactive technologies” for emotion regulation training identified 65 peer-reviewed papers (including 4 RCTs and 28 controlled studies) that met their inclusion criteria [15]. Across these, they identified several theoretical models underpinning the DHIs, including biofeedback and neurofeedback as well as psychotherapeutic models such as CBT, the dual pathway model, emotion-focused therapy, attachment theory, and positive psychology, with the majority (72%) of DHIs being CBT-based.

### Emotion Regulation Interventions for Adolescents With ADHD

The lack of evidence-based DHIs for emotion regulation in adolescents with ADHD is reflective of a broader lack of interventions for emotion regulation in adolescents with ADHD. For example, one recent review [5] examined the literature on nonpharmacological emotion regulation interventions for children and adolescents and found only 5 RCTs, 1 quasi-experimental study, 4 open-label uncontrolled studies, and 2 ongoing studies without results. These studies evaluated a range of interventions but most were based on behavioral therapy or CBT (with or without parent training). Though results generally suggested that these types of nonpharmacological interventions could improve emotion regulation in young people with ADHD, they noted several important limitations with the study designs such as small sample sizes, lack of randomization, issues with emotion regulation measurement, and lack of evaluation of medium- to long-term effects. In addition, only 1 study was focused on adolescence, and the predominant focus was on the age range of 6-13 years. A few additional studies have been published since the review with promising findings [33,34]. For example, the “Regulating Emotions Like an Expert” program aims to improve emotion regulation and decrease family conflict by teaching both adolescents and their caregivers emotion regulation strategies while additionally providing conflict management and communication skills. It draws on CBT and psychoeducation and was associated with adolescent improvements in emotion regulation in a small-scale evaluation. However, overall evidence on what works for emotion regulation in adolescents with ADHD remains sparse. With only a few exceptions, there is a lack of interventions for emotion regulation that have been designed specifically for adolescents with ADHD [33]. Given this lack of tailoring to adolescents with ADHD, emotion regulation interventions that are currently available are not likely to be optimized for this group.

### Emotion Regulation Interventions for Adolescents

Despite the limitations in evidence on emotion regulation interventions for adolescents with ADHD, the transdiagnostic nature of emotion regulation means that relevant insights may come from the broader literature on emotion regulation interventions for adolescents. Reviews of this literature suggest that overall emotion regulation can be effectively improved through nonpharmacological interventions, with the strongest effects seen in clinical samples [35-38]. For example, in a recent meta-analysis of emotion regulation interventions focused specifically on adolescents, the pooled effect size for controlled studies was Hedge *g*=0.19 and was *g*=0.29 for pre-post studies [35]. Included studies used a range of approaches and combinations, including mindfulness, dialectical behavior therapy (DBT), psychodynamic therapy, emotion-focused cognitive behavioral therapy (ECBT), psychoeducation, and behavioral activation to target a variety of emotion regulation components, and were delivered in individual and group formats (eg, in school settings), in some cases, involving parents as well as the adolescents themselves. Only 1 study included in the review was focused on adolescents with ADHD (the Regulating Emotions Like an Expert intervention discussed earlier), and only a handful of others focused on neurodivergent adolescents (all studies in samples of individuals with autism) [39-41].

One study [36] also meta-analyzed the literature on emotion regulation interventions for adolescents but additionally used a 3-level meta-analysis to explore which intervention elements were associated with stronger intervention effects. They coded 75 different intervention elements (within 15 categories) and found that interventions that included setting goals for treatment and *psychoeducation about acceptance* had significantly higher effect sizes, whereas the element *social skills training, unspecified* was associated with significantly worse effects. *Setting goals for treatment* refers to setting goals for contact with the adolescent as well as setting realistic session-by-session goals. *Psychoeducation about acceptance* refers to providing information on what it means to accept thoughts or emotions and how this can be an alternative strategy to control or avoidance. Several other elements were not significant but were associated with effect size differences greater than >0.20 and therefore may also represent important active ingredients. These included discussion of challenging emotional situations, alternative actions to emotional avoidance, downregulation of negative emotions, exposure to emotions, self-exploration or self-monitoring of thoughts and feelings, unspecified, teach cognitive flexibility reappraisal, psychoeducation about treatment or treatment element or technique, psychoeducation about self-esteem and self-worth, psychoeducation about stress, evaluating consequence of behaviors, and lifestyle, unspecified. These elements could be considered for ADHD-specific DHIs for emotion regulation; however, some elements (eg, discussion of challenging emotional situations) would be more difficult to translate to this delivery mode than others (eg, psychoeducation about acceptance).

## Intervention Approaches and Components

### Overview

The “Existing (Digital Health) Interventions Relevant for Emotion Regulation in Adolescents With ADHD” section illustrates the range of therapeutic approaches that have been used in interventions to support emotion regulation in adolescents with and without ADHD. In this section, we discuss key approaches in more detail to illustrate and compare the different ways in which existing approaches address emotion regulation difficulties. Of note, there are shared or similar features across many approaches, though the labeling of these features may differ by therapeutic framework.

### Cognitive Behavioral Therapy

CBT (also sometimes referred to as “behavioral therapy” in an ADHD context) is a versatile therapeutic approach that targets cognitions and underlying schema while facilitating adaptive behavioral strategies. CBT is commonly used in emotion regulation interventions for adolescents without ADHD [35]. In addition, though research on emotion regulation outcomes following CBT in ADHD is scarce, it is frequently used and has been widely evaluated in the treatment of adolescents with ADHD to target various other outcomes, including primary ADHD symptoms (inattention and hyperactivity or impulsivity), organization skills, and social skills [42]. The literature on the effectiveness of CBT in ADHD has been characterized as heterogeneous and difficult to summarize; however, the overall indication is that it is effective in improving primary symptoms and functional outcomes [42,43]. A recent meta-analysis, for example, found a pooled effect size for CBT interventions in children and adolescents with ADHD of *g*=0.80 [44], and another review discussed positive effects on a range of outcomes that are meaningful for people with ADHD [42], including demonstrations of incremental benefits when added to medication. CBT has been adapted and combined with several other approaches to provide better tailoring to the specific issue of emotion regulation. The most widely researched of these approaches are discussed below. As noted earlier, there is a considerable precedent for the incorporation of CBT skills in DHIs for emotion regulation, suggesting that DHIs represent a viable approach for the delivery of CBT-derived interventions [15].

### Emotion-Focused CBT

Researchers have argued for the benefits of a greater focus on emotion in CBT, where the outcome of interest is improved emotional functioning [45]. As a result, a variant of CBT, ECBT that includes core CBT components as well as content that is specifically focused on the regulation of a variety of emotions (eg, sadness, guilt, and anger), has been developed to address this gap [45]. In face-to-face therapy, ECBT sessions may involve content around identifying emotions, causes and consequences of emotions and associated bodily sensations, the regulation of emotions, and emotional exposure tasks. Despite the face validity of this increased focus on emotions, however, findings on the relative advantages of ECBT for emotion regulation are somewhat mixed. One study in adolescents without ADHD found that ECBT was associated with greater improvements in emotion regulation outcomes compared to both no treatment and CBT; however, another study found that ECBT and CBT were associated with similar improvements in emotion dysregulation and adaptive emotion regulation strategy use [45,46]. Overall, there has been very little evaluation of ECBT with adolescents, and none we could identify with adolescents with ADHD specifically. Future research could therefore explore fruitful adaptations to this group, for example, exploring the regulation of positive as well as negative emotions, given that dysregulation of the former may also represent a source of impairment for adolescents with ADHD [47].

### Unified Protocol

The “unified protocol” [48] is a CBT-based manualized transdiagnostic intervention for emotion dysregulation, which may also be considered a form of ECBT due to its strong focus on emotions. The approach has empirical support for improving symptoms of internalizing problems [49] and is available in a form adapted for children and adolescents [50]. It typically includes modules focusing on different aspects of emotion regulation (eg, cognitive reappraisal, tolerance, and reducing the use of avoidance), accompanied by psychoeducational materials, motivational interviewing or enhancement, and review and relapse prevention. A key goal is to promote the use of more adaptive emotion regulation strategies and reduce the use of more maladaptive strategies. This has strong relevance for ADHD, which has been associated with the use of less adaptive strategies and more maladaptive strategies [51]. It also aims to support the development of related competencies such as awareness or acceptance of emotional experience, flexible modulation of emotional intensity and duration, and willingness to experience emotions within everyday life. Future research could explore how to adapt the intervention for this group, such as offering a greater focus on the aspects of emotion regulation (eg, affective lability) and its impacts (eg, peer problems) that are most pertinent for adolescents with ADHD [52].

### Rumination-Focused CBT

Rumination is often classified as a maladaptive emotion regulation strategy characterized by a repetitive style of negative thinking that can maintain or escalate negative affect due to a focus on one’s negative emotions and the causes and consequences of them, without engaging in problem-solving [53]. Consistent with emotion regulation more generally, evidence suggests that it can act as a mediator in the relations between ADHD and mental health outcomes [54,55]. Rumination-focused cognitive behavioral therapy (RF-CBT) is based on differentiating between adaptive and maladaptive styles of repetitive thinking and uses functional analysis to examine the functions of ruminations [56]. It aims to equip patients with skills and strategies to reduce rumination, such as fostering more concrete thinking and developing “if ... then” implementation plans in response to rumination “warning signs.” RF-CBT has been found to be helpful in reducing and preventing emotional disorder symptoms [57]; however, it has not been specifically evaluated as a treatment for adolescents with ADHD. In terms of its potential for adaptation in DHIs, self-help versions are available, providing proof-of-concept for delivery without clinician input [58].

### Mindfulness-Based Interventions and Mindfulness-Based Cognitive Therapy

Mindfulness-based interventions have been proposed for strengthening emotion regulation skills, including in young people with ADHD [59]. Though available in different forms, a common theme of mindfulness-based interventions is that they encourage attending to the current moment with acceptance and without judgment. This may be achieved through daily meditation and self-awareness activities, sometimes combined with CBT techniques in mindfulness-based cognitive therapy (MBCT) [59]. Recent meta-analyses have suggested mixed findings on the effects of mindfulness-based interventions for adolescents [35]. For example, one study showed MBCT-associated improvements in emotional awareness but not in nonacceptance of emotions, impulse control, or regulation [60], and of the various studies that reported evaluations of the Learn to Breathe intervention, some showed no effect and others reported effects on a subset of emotion regulation outcomes only [61-66]. The evidence on the efficacy of mindfulness-based interventions on emotion regulation for adolescents with ADHD is scarce and inconsistent, based on small-scale studies, and has not focused on emotion regulation as an outcome [33]. In terms of the viability of a DHI based on this approach, in addition to the strong precedent for DHI delivery of CBT-derived interventions, mindfulness apps for emotion regulation have previously been successfully developed and found to be feasible for use in adolescents [67].

### Acceptance and Commitment Therapy

Acceptance and commitment therapy (ACT) is another therapeutic approach that, though not necessarily its primary focus, addresses emotion regulation in various ways such as teaching emotional acceptance over suppression and avoidance, encouraging engagement with value-consistent behaviors despite negative affect, and promoting psychological flexibility. As such, ACT has informed or been incorporated into interventions targeting emotion regulation in patient groups such as those experiencing nonsuicidal self-injury [68] as well as vulnerable young people [69] and students [70]. There are, however, very few studies that have used ACT in ADHD. A recent scoping review identified only 6 studies from 2015 and none that examined its use in directly addressing emotion regulation in ADHD [71]. However, previous studies have demonstrated how ACT can be translated into a DHI format [72], as well as establishing acceptability and feasibility when used in patient groups such as those with generalized anxiety disorder [73] or eating disorder [72]. This suggests that despite the lack of research into ACT in ADHD, it holds potential for delivery in a DHI format.

### Dialectical Behavior Therapy

DBT was originally developed for patients with borderline personality disorder but has since been used across disorders to improve emotion dysregulation and related outcomes such as suicidality and self-harm [74,75]. DBT has also been used with adults with ADHD [76]. In common with CBT, DBT aims to address maladaptive cognitive and behavioral processes; however, it also has a strong emphasis on mindfulness and acceptance, thus also showing some convergence with the principles of MBCT and ACT. DBT has shown some promising findings for improving emotion regulation and other outcomes in adults with ADHD [77]; however, one recent RCT showed no clear evidence of benefit for emotion regulation in adults with ADHD [78], despite being associated with improvements in other outcomes, including emotional disorder symptoms. More generally, a recent systematic review not specifically focused on populations with ADHD found a lack of evidence for a positive effect of interventions based on DBT (primarily interventions that used the skills acquisition components alone) on emotion regulation, despite this being a core hypothesized mechanism of action [79]. In addition, while DBT has been adapted for use in adolescents [80], there is a lack of DBT studies with adolescents with ADHD specifically. In terms of the viability of delivering DBT via DHIs, a recent review paper synthesized the literature on mobile apps using DBT and identified 21 apps (2 of which were specifically aimed at adolescents). Of these, 71% were scored as showing “acceptable” usability, suggesting that DHI delivery of DBT is feasible [81].

### Adjunctive Components

Components of other therapeutic approaches that can be used as adjuncts or otherwise combined with other approaches have been explored to support emotion regulation in adolescents with ADHD. Psychoeducation, such as providing information about emotion regulation or dysregulation, is very commonly included in emotion regulation interventions for young people both with and without ADHD [33,34,82]. While there is limited evidence for the efficacy of psychoeducation for improving emotion regulation as a stand-alone intervention [83], there are several studies suggesting the efficacy of interventions that include a psychoeducation component [5]. In addition, the common elements analysis [36] discussed earlier identified several psychoeducation-based elements to be associated with increased intervention effect sizes. Taken together, psychoeducation, in combination with other approaches, appears to be a valuable component of emotion regulation interventions. For adolescents with ADHD, it is likely to be crucial to deliver psychoeducation in engaging formats (using images or videos and minimizing text), potentially broken down into smaller chunks.

Music therapy has also been proposed as an adjunct in emotion regulation interventions. One study [84] reported preliminary evidence that a combined CBT and music-based intervention improved emotion regulation skills in adolescents with ADHD and was considered high in acceptability by participants. Music therapy has also been trialed as an emotion regulation intervention in general at-risk samples of young people, with some promising findings [85,86]. Given that music-based emotion regulation interventions are also feasible to be delivered via DHIs [87], this merits further investigation as an intervention component for DHIs for adolescents with ADHD.

Symptom monitoring is common and well-accepted in DHIs [88]. A recent study reported the positive effects of smartphone-based symptom monitoring in adolescents with ADHD [89]. However, to our knowledge, this has not been evaluated in relation to emotion regulation specifically and requires future research to determine its promise and how to optimize its implementation in this context.

Finally, it is important to consider the role of parents and caregivers in supporting the treatment of adolescents with ADHD and the implications of this for emotion regulation DHIs. Many effective interventions for adolescents with ADHD include a parent or a dyadic component or leverage parents to, for example, support the young person by motivating, reminding, and monitoring them in practicing and generalizing their skills. Parent or caregiver involvement in such interventions is seen as an important aspect of their success [90,91]. Indeed, previous research has suggested that the involvement of parents or caregivers in interventions for young people with ADHD, either as an intervention target or to reinforce the intervention received by the young person, improves outcomes for the young people [92,93]. However, parents or caregivers can also be a source of barriers to accessing and engaging with treatment in adolescents with ADHD. For example, studies have identified low parent motivation as a treatment barrier for adolescents with ADHD, as well as low parent adherence during treatment [90,94]. For example, in an analysis of psychosocial treatment engagement barriers for adolescents with ADHD, there was a 69.4% prevalence of parental failure to consistently monitor their teen’s skills use and a 49.6% prevalence of intrusive behaviors that undermined the autonomous practice of skills by youths [90]. This suggests that while parent or caregiver involvement could enhance the effects of DHIs for adolescents with ADHD, they should not depend on parental involvement. This could be operationalized in terms of providing optional parent or caregiver-oriented materials that could be used to support the young person’s learning and practice as well as supporting parent or caregiver-adolescent relationship aspects of emotion regulation (including fostering effective interpersonal emotion regulation). It could additionally involve identifying ways in which the DHI might serve some of the roles that parents or caregivers typically take on in supporting treatment engagement, such as notifications to complete tasks within the app, reminders to practice skills outside it, and offering rewards for successful completion.

## Other Design, Development, and Evaluation Considerations

### DHIs Informed by the Latest Developments in Emotion Regulation and Research

It is critical that DHIs are informed by—in combination with user and clinical input—research evidence over the lifecycle of their development and evaluation. Emotion regulation research is an active area of inquiry in which there continue to be new developments that can inform interventions. For example, recent research has seen a relative shift in focus from identifying adaptive versus maladaptive strategies to investigating the flexibility with which individuals deploy strategies [95]. Similarly, the universal value of “adaptive” strategies such as cognitive reappraisal has been more recently questioned [96]. New measures are being developed within ADHD research, reflecting a need for ADHD-specific measures and also addressing the lack of focus on strategies beyond reappraisal and suppression captured by dominant existing measures [97]. For example, in addition to reappraisal and suppression, the recently developed Comprehensive Emotion Regulation Inventory measures emotional reactivity, situational selection, modification, attentional deployment, implementation, and negative impact [97]. More attention is also now being paid to inter- as well as intrapersonal emotion regulation strategies [98]. Interpersonal emotion regulation strategies refer to the use of others in the service of regulating emotions, whereas intrapersonal regulation strategies are those such as suppression and reappraisal that occur within an individual. New evidence along these and other lines of research in emotion regulation should be reflected in intervention approaches, and interventions should adopt a development and evaluation approach (such as “trials of intervention principles”) that is agile enough to respond to new evidence as well as technological advances [99].

### Promoting Engagement With DHIs

A number of discussions of DHIs have noted significant challenges in the sustained engagement of users, for example, prominent authors in the field have noted that “user engagement with digital mental health is the new primary challenge in realizing the full potential of scalable and accessible care” [100]. One analysis of mental health app retention, for example, found a median 15-day retention rate of only 3.9% [101]. Prompted by the growing recognition of these issues, there has been an increased research focus on the barriers and facilitators to DHI engagement and associated engagement strategies, including a number of recent reviews on the topic [100,102-106].

An early analysis of the contributing factors to low engagement [105] proposed several key factors that may limit engagement, including that DHIs are not sufficiently designed with users in mind (eg, are not enjoyable to use), do not solve problems that users care about, and are unhelpful in crises and emergencies. In addition, there were concerns around privacy and trust. One recent comprehensive review examined the barriers and facilitators to mental health DHI engagement, identifying 208 eligible papers for inclusion [102]. Based on the information extracted in the included studies, they classified factors in terms of those related to the user, program or content offered by the intervention, and the technology and implementation environment. In terms of users, they found that women were more likely to engage with DHIs than men and that mental health symptoms sometimes made engagement with DHIs more challenging. Positive beliefs about mental health help-seeking and technology, digital health literacy, and positive experiences with mental health services and technology facilitated engagement. DHI engagement was additionally facilitated by people being able to integrate DHI use into their daily lives; it was facilitated when the DHI content was credible, of an appropriate length, and customizable to the user; and it was facilitated if users understood the data shown by the DHI and if they knew how to use the DHI. Guided DHIs (including via automated reminders) generally yielded higher engagement than unguided interventions, as did DHIs that facilitated social connection. Engagement was higher when participants had a sense that the platform was private and anonymous and if they felt that the people close to them thought they should use DHIs. Finally, technical issues were a barrier to engagement, whereas training people to use the DHI was a facilitator.

While these factors provide general information about DHI engagement, it will be important to understand the specific barriers and facilitators of DHI engagement experienced by adolescents with ADHD. For example, difficulties with sustaining attention, increased forgetfulness, lower frustration tolerance, high novelty seeking, and differences in reward sensitivity and delay discounting (eg, a preference to receive a smaller reward sooner over a larger reward later) compared to non-ADHD DHI users may be particularly pertinent characteristics to consider when mapping factors influencing engagement [107-109].

In terms of engagement strategies, one review of emotion regulation DHIs [15] documented the specific engagement strategies used in emotion regulation DHIs. Approaches included the use of gamification (the integration of game mechanics to improve adherence, motivation, and enjoyment), multimodal representations of emotional experience, and storytelling. For example, one DHI used a digital storytelling method, in which a grumpy pirate is searching for treasure [110]. During the course of the narrative, users are encouraged to relive their feelings in a safe setting, supported by their caregiver. The review also highlighted how personalization may be used in emotion regulation DHIs, facilitated, for example, by using machine learning to predict emotional states and user customization features.

Reviews of DHIs in ADHD have not generally discussed engagement strategies in depth; however, an inspection of primary papers suggests that approaches used include the use of high-quality engaging graphics, reward loops, personalization (including adaptive personalization of difficulty level), exercises that elicited more active engagement with the app, gamification, prompts or reminders, feedback on progress and compliance, co-design with young people, and the ability to schedule chat sessions [111-118]. For example, the project EVO DHI, which targets neurocognitive functioning, uses a consumer-grade video game–type format to appeal to young people [111], while a DHI designed to improve medication adherence successfully used text reminders to encourage the target behavior [119]. Despite the fledging status of this area of research, several authors have noted the promise of strategies such as gamification to overcome intervention adherence difficulties in young people with ADHD [120,121]. Taken together, while there is limited evidence on which specific strategies are optimal for DHIs for emotion regulation in adolescents with ADHD, the importance of developing and evaluating [102] engagement strategies is clear.

The development of engagement strategies is one aspect of DHIs that may particularly benefit from a coproduction approach with users themselves. A common theme in analyses of barriers to engagement relates to issues deriving from a lack of user involvement from the earliest stages of DHI development. One analysis [105], for example, noted that users can provide critical feedback that helps make sure that a DHI addresses real-world problems that matter to users; yet, a minority of DHIs has the necessary level of user involvement throughout the process. A recent review of DHIs for ADHD [24] also noted that there were few DHIs in the included studies that were designed based on user-centered participatory and ability-based design approaches. One notable exception can be seen in the series of participatory workshops conducted by the authors of a smartwatch-based intervention to support self-regulation among young people with ADHD [122]. These workshops explored topics such as what features the users would like to see in the intervention and what “engagement strategies” would help adherence, using discursive methods and engaging activities.

### DHI Evaluation

Despite large numbers of ADHD DHIs being available, only a small minority are evidence-based [26]. This is an important issue because there is an obvious ethical obligation for interventions used by adolescents with ADHD to be effective and not cause harm. This issue also impacts dissemination and engagement with DHIs because users and those who may recommend DHIs (eg, clinicians) may feel a lack of trust in the effectiveness of DHIs [105]. Best practices for DHI evaluation broadly follow principles and processes similar to those for intervention evaluation in general; however, there are also some unique considerations in assessing the effectiveness, usability, and scalability.

A comprehensive evaluation should include several steps. First, studies that focus on the thorough assessment of the intervention’s design, functionality, and user interface, ensuring that it aligns with the targeted health goals and user needs should be conducted. Second, the usability and feasibility of DHIs should be tested with the aim of understanding how easily the DHI can be integrated into existing health care systems and routines [123]. Finally, rigorous methodologies, such as RCTs, should be applied to evaluate the impact on patient or client outcomes. DHI evaluations also need to consider DHI-specific challenges related to data security and privacy concerns. As the DHI and the evaluation fields continue to evolve, DHI developers need to be aware of and incorporate the latest developments in DHI evaluation best practice [124].

## Recommendations

The recommendations are as follows: (1) the limited evidence available suggests that there are several promising therapeutic approaches that could inform emotion regulation DHIs for adolescents with ADHD, including emotion regulation–focused CBT variants (eg, ECBT, unified protocol, and RF-CBT), DBT, and ACT; (2) users should be involved in DHI development from the very beginning; (3) the embeddedness of smartphone use in daily life to promote practicing skills in real-life situations could be leveraged to promote generalizable improvements; (4) strategies to promote sustained engagement should be co-designed with users and built into DHIs; and (5) DHIs should take a development and evaluation approach that allows them to be responsive to the latest evidence in the active area of research into ADHD emotion regulation.

## Conclusions

There is limited direct evidence to inform the design of DHIs to support emotion regulation in adolescents with ADHD. However, there is a strong precedent for the use of DHIs with young people with ADHD and for the use of DHIs to deliver emotion regulation interventions in populations without ADHD. There are several promising therapeutic approaches that could underpin such DHIs, including emotion regulation–focused CBT approaches and components (eg, ECBT, RF-CBT, and unified protocol), DBT, ACT, psychoeducation, music therapy, and symptom tracking; however, much further work is required to gain a better understanding of whether they can be used to underpin effective ADHD-tailored emotion regulation DHIs.

In terms of the DHI development process, involving users across the lifecycle of development and co-designing strategies to support sustained engagement will be critical. As emotion regulation in ADHD is an active area of research, DHIs will need to be able to be responsive to the latest developments. Finally, we speculate that the greatest benefit of emotion regulation DHIs may be derived, where they are leveraged to promote the practice and use of skills in the course of daily life (eg, via reminders throughout the day to use the skills learned).

## Abbreviations

ACT: acceptance and commitment therapy
ADHD: attention-deficit/hyperactivity disorder
CBT: cognitive behavioral therapy
DBT: dialectical behavior therapy
DHI: digital health intervention
ECBT: emotion-focused cognitive behavioral therapy
MBCT: mindfulness-based cognitive therapy
RCT: randomized controlled trial
RF-CBT: rumination-focused cognitive behavioral therapy
